# ASO Author Reflections: 30 Years of Esophagectomy

**DOI:** 10.1245/s10434-020-09208-9

**Published:** 2021-03-18

**Authors:** A. W. Phillips, S. K. Kamarajah, S. M. Griffin

**Affiliations:** 1 Northern Oesophagogastric Unit, Newcastle upon Tyne NHS Foundation Trust, Newcastle upon Tyne, UK; 2 School of Medical Education, Newcastle University, Newcastle upon Tyne, UK

## Past

The past 30 years have seen a huge change in the management and outcomes of patients with esophageal cancer. There has long been a perception of dismal outcomes for those patients requiring esophagectomy, with surgery associated with high levels of mortality, long-standing morbidity, and a prolonged hospital stay.^1^

Surgery has always been the cornerstone of curative treatment, allowing removal of the cancer. Lymphadenectomy has been a contentious component to surgery; some suggest that it confers no benefit but adds additional morbidity. There has been increased understanding of the need for lymphadenectomy and the importance of neoadjuvant treatment conferring optimal outcomes.

## Present

The present study evaluated the outcomes of patients with both adenocarcinoma and squamous cell carcinoma undergoing curative procedures during a 30-year period from a single, high-volume center. It demonstrates a huge improvement in overall survival and correlates this with a number of interventions.^2^

Predictably neoadjuvant chemotherapy and chemoradiotherapy have had the biggest impact on long-term survival. However, the implementation of an enhanced recovery pathway has drastically reduced inpatient stay after surgery. Presently, patients receive an increasingly individualized treatment pathway, with endoscopic treatment of early lesions, thoracoscopic surgery, and neoadjuvant and adjuvant regimens employed as required. Close contact with enhanced recovery team members ensures mobility, nutrition, and recovery after surgery are provided in an optimal fashion.

## Future

Despite the huge improvements, overall 5-year survival still only approaches 50%. The future may permit further tailoring of treatment with oncological therapies specific to the genetics of the particular tumor. Furthermore, surgical techniques are evolving. More research is needed into the use of endoscopic therapy for anything but the earliest cancers, and robotic technology is making technically demanding surgery more straightforward, which should confer morbidity and mortality advantages of patients. Prehabilitation to ensure patients are fit for surgery is starting to get integrated into patient pathways, and a more structured rehabilitation pathway, which builds on the enhanced recovery within the immediate postoperative period will help to improve patients’ quality of life. Much can still be done to improve the outcomes for our patients.
